# Snail regulates Hippo signalling-mediated cell proliferation and tissue growth in *Drosophila*

**DOI:** 10.1098/rsob.210357

**Published:** 2022-03-09

**Authors:** Xiang Ding, Zhuojie Li, Kai Peng, Rui Zou, Chenxi Wu, Gufa Lin, Wenzhe Li, Lei Xue

**Affiliations:** ^1^ Institute of Intervention Vessel, Shanghai 10th People's Hospital, Shanghai Key Laboratory of Signaling and Disease Research, School of Life Science and Technology, Tongji University, Shanghai, People's Republic of China; ^2^ College of Traditional Chinese Medicine, North China University of Science and Technology, Tangshan, Hebei, People's Republic of China; ^3^ Key Laboratory of Spine and Spinal Cord Injury Repair and Regeneration of Ministry of Education, Orthopaedic Department of Tongji Hospital, School of Life Sciences and Technology, Tongji University, Shanghai, People's Republic of China; ^4^ Zhuhai Precision Medical Center, Zhuhai People's Hospital, Zhuhai Hospital Affiliated with Jinan University, Zhuhai, Guangdong, People's Republic of China; ^5^ National Clinical Research Center for Interventional Medicine, Shanghai 10th People's Hospital, Tongji University, Shanghai, People's Republic of China

**Keywords:** snail, Hippo pathway, tissue growth, cell proliferation, *Drosophila*

## Abstract

Snail (Sna) plays a pivotal role in epithelia-mesenchymal transition and cancer metastasis, yet its functions in normal tissue development remain elusive. Here, using *Drosophila* as a model organism, we identified Sna as an essential regulator of Hippo signalling-mediated cell proliferation and tissue growth. First, Sna is necessary and sufficient for impaired Hippo signalling-induced cell proliferation and tissue overgrowth. Second, Sna is necessary and sufficient for the expression of Hippo pathway target genes. Third, genetic epistasis data indicate Sna acts downstream of Yki in the Hippo signalling. Finally, Sna is physiologically required for tissue growth in normal development. Mechanistically, Yki activates the transcription of *sna*, whose protein product binds to Scalloped (Sd) and promotes Sd-dependent cell proliferation. Thus, this study uncovered a previously unknown physiological function of Sna in normal tissue development and revealed the underlying mechanism by which Sna modulates Hippo signalling-mediated cell proliferation and tissue growth.

## Graphical abstract

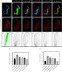

## 1. Introduction

Tissue growth and organ size are controlled by coordinated regulation of cell number and cell size, while maintenance of cell number depends on the balance of cell death and proliferation. The Hippo pathway, first identified in *Drosophila*, enables evolutionarily conserved signalling that regulates tissue growth and organ size in animal development [1]. The core components consist of the upstream kinase Hippo (Hpo), which phosphorylates and activates the downstream kinase Warts (Wts) [2]. Activated Wts phosphorylates the transcription cofactor Yorkie (Yki) [3], which is retained in the cytoplasm by physical interaction with the adaptor protein 14-3-3 [4]. When the Hippo pathway is inactive, unphosphorylated Yki enters the nucleus to form a complex with the transcription factor Scalloped (Sd), which activates the expression of target genes involved in the control of cell growth, proliferation, survival and metabolism [5–7]. Hippo signalling also plays critical roles in stem cell renewal and differentiation, innate immunity and tumorigenesis [8–11]. Although more than 30 components/regulators of the Hippo pathway besides the core kinase cascade have been characterized over the past decade [12], additional factors that modulate Hippo signalling-mediated tissue growth remain to be elucidated.

*snail* (*sna*) encodes an evolutionary conserved zinc finger transcription factor [13], which was first characterized in *Drosophila* as a critical regulator of embryonic mesoderm formation [14] and was late reported to play a key role in tumour invasion and metastasis, especially in epithelial-mesenchymal transition (EMT) [15,16]. Sna acts as a transcriptional repressor regulating a large number of genes involved in the EMT process [17,18]. For instance, the overexpression of SNAI1 in tumour cell lines promotes tumour metastasis [19,20]. Besides its well-known functions in embryo development and tumour metastasis, other studies suggest that Sna also plays important roles in regulating multiple biological processes including cell proliferation, cell differentiation and cell death [21–26]. However, the mechanism by which Sna regulates tissue homeostasis remains not fully understood. Due to the low redundancy, *Drosophila* is an excellent model system to investigate the physiological functions of Sna in tissue/organ development.

In this study, we identified Sna as a crucial modulator of Hippo signalling-mediated tissue growth in *Drosophila* development. Loss of *sna* inhibits, while overexpression of Sna promotes, Hippo signalling-mediated cell proliferation and tissue growth. In addition, Sna is physiologically required for tissue growth in normal development. The genetic epistasis analysis indicates that Sna acts downstream of Yki to promote target genes expression and cell proliferation. Mechanistically, Yki activates *sna* transcription, while elevated Sna binds to Sd and promotes Sd-dependent cell proliferation. In conclusion, our results identified Sna as an essential regulator of the Hippo pathway and revealed the underlying mechanism by which Sna modulates Hippo signalling-mediated cell proliferation, tissue growth and tumour progression.

## 2. Results

### 2.1. Loss of *sna* suppresses Hippo signalling-mediated tissue overgrowth

**Figure 1.**
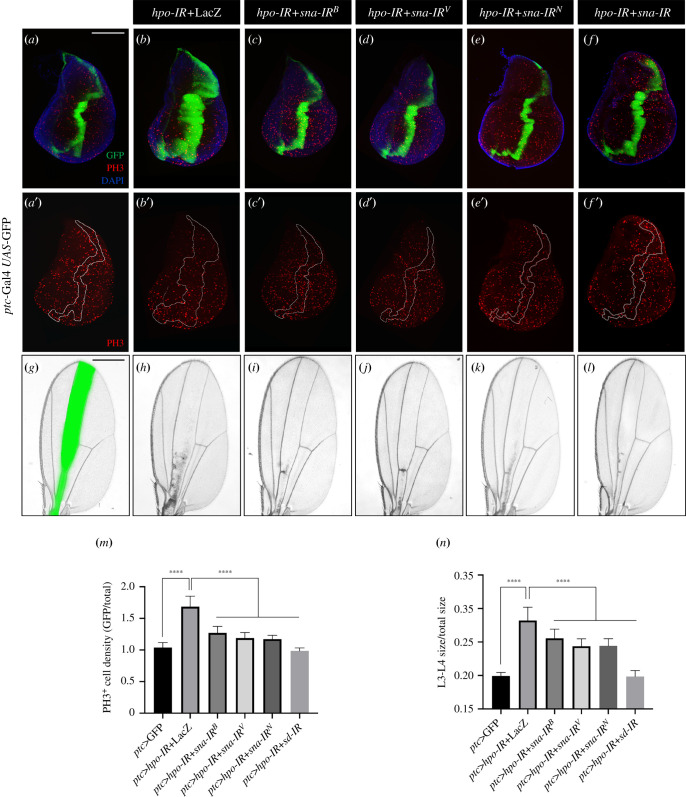
The loss of *sna* suppresses *hpo* depletion-induced cell proliferation and tissue overgrowth. (*a*–*f*) Fluorescence micrographs of third-instar larval wing discs stained with anti-pH3 (red) are shown. Compared with the *ptc* > GFP control (*a*,*a′*), *hpo* knockdown increases the width of *ptc*-stripe (*b*) and pH3-positive cell density within the stripe (*b′*), both of which are significantly suppressed by expressing three independent *sna RNAi* (*c*,*c′*, *d*,*d′*, *e* and *e′*), *sd RNAi* serves as a positive control (*f*,*f′*). (*g*–*l*) Light micrographs of *Drosophila* adult wings are shown. Compared with the *ptc*-Gal4 control (*g*), the expression of *hpo-IR* + LacZ causes an expanded L3-L4 area (*h*), which is suppressed by depletion of *sna* (*i*–*k*) or *sd* (*l*). (*m*) Quantification of PH3-positive cell density ratio for GFP region/total region (left to right: *n* = 10, *n* = 9, *n* = 8, *n* = 9, *n* = 10, *n* = 7). (*n*) Quantification of size ratio for L3-L4 area/total area (left to right: *n* = 10, *n* = 15, *n* = 24, *n* = 24, *n* = 19, *n* = 20). One-way ANOVA was used to compute *p*-values, ****p < 0.0001. Scale bar: 100 µm in (*a*–*f*), 250 µm in *g*–*l*.

To investigate the genetic interaction between Sna and Hippo pathway, we first checked whether Sna is required for Hippo signalling-mediated overgrowth. Compared with the control (figure 1*a*), inactivated Hippo signalling by depleting *hpo* along the A/*P* compartment boundary in third-instar larval wing discs driven by *ptc*-Gal4 robustly increased the width of *ptc*-expressing stripe (figure 1*b*) [27]. This phenotype was significantly suppressed by expressing three independent *sna-IR* lines that target distinct regions of the *sna* transcript [23] (figure 1*c*–*e*), while *sd-IR* was included as a positive control (figure 1*f*). Hippo pathway inactivation promotes tissue overgrowth mainly through accelerated cell proliferation [2,28]. Consistently, the depletion of *hpo* promoted cell proliferation in the corresponding region detected by increased anti-PH3 staining, which was dramatically suppressed by knocking-down *sna* and *sd* (figure 1*a*’–*f*’,*m*; electronic supplementary material, figure S1). In addition, *ptc* > *hpo-IR +* LacZ animals displayed enlarged area between L3 and L4 in the adult fly wings, which was also suppressed by depleting *sna* and *sd* (figure 1*g*–*l*,*n*). A quantitative reverse transcription polymerase chain reaction (RT-qPCR) assay was performed to assess the knockdown efficiencies of the three *sna* RNAi lines (electronic supplementary material, figure S2). By contrast, *ptc* > *sna-IR* did not cause any obvious change in cell proliferation or tissue growth (electronic supplementary material, figure S3). Furthermore, *hpo-IR* and *hpo-IR* + *sna-IR* did not affect cell death or cell size (electronic supplementary material, figure S4). Collectively, these results indicate that Sna is required for *hpo* depletion-triggered cell proliferation and tissue overgrowth.

**Figure 2.**
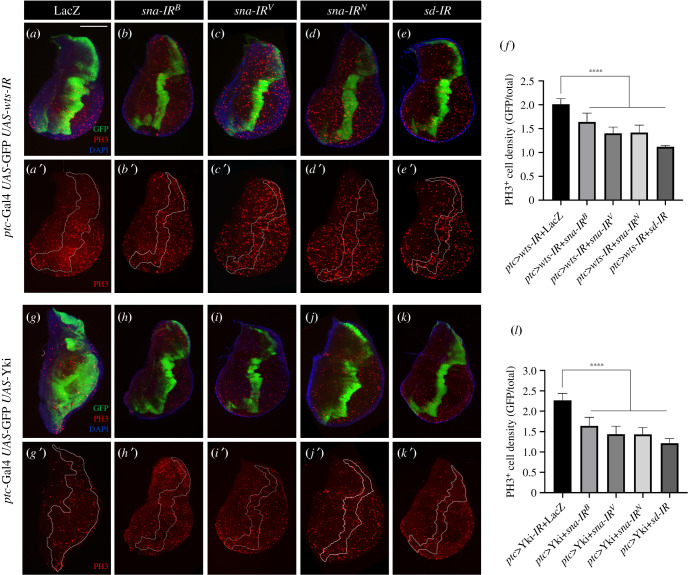
The depletion of *sna* suppresses Hippo pathway-mediated cell proliferation. (*a*–*e*, *g*–*k*) Fluorescence micrographs of third-instar larval wing discs stained with anti-pH3 antibody (red) are shown. *ptc > wts-IR* or *ptc* > Yki promotes tissue overgrowth (GFP in (*a*,*g*)) and increases pH3-positive cell density (red in *a′*,*g′*), both of which are suppressed by expressing *sna-IR* (*b*–*d*, *h*–*j*) or *sd-IR* (*e*, *k*). (*f*,*l*) Quantification of pH3-positive cell density ratio for GFP region/total region. (*f*) Left to right: *n* = 14, *n* = 10, *n* = 12, *n* = 12, *n* = 10. (*l*) Left to right: *n* = 17, *n* = 12, *n* = 12, *n* = 9, *n* = 8. One-way ANOVA was used to compute *p*-values, ****p < 0.0001. Scale bar: 100 µm in (*a*–*e*), (*g*–*k*).

To dissect the mechanism by which Sna modulates Hippo signalling, we performed genetic epistasis analysis between Sna and Hippo pathway core components. *warts* (*wts*) encodes a serine/threonine kinase acting downstream of Hpo, and *ptc > wts-IR* produced similar phenotypes as that of *ptc > hpo-IR*, including expanded *ptc*-stripe and increased PH3-positive cell density [29]. Both phenotypes were suppressed by expressing *sna-IR* or *sd-IR* (figure 2*a*–*f*), suggesting Sna modulates Hippo pathway downstream of Wts.

Impaired Hippo signalling leads to the nuclear translocation of Yki and promotes Yki-dependent cell proliferation [3,30]. Ectopic expression of Yki dramatically promoted tissue overgrowth and cell proliferation [31], which were partially suppressed by depleting *sna* or *sd* (figure 2*g*–*l*). In addition, expressing an activated form of Yki (Yki^S168A^) by *en*-Gal4 promoted tissue overgrowth and cell proliferation in the *P*-compartment of wing discs, both of which were significantly suppressed by knockdown of *sna* or *sd* (electronic supplementary material, figure S5).

While *sd* is reported to be specifically expressed in the wing pouch of third-instar larvae, we noticed that *sd-IR* suppressed Hippo-Yki signalling-induced tissue overgrowth and cell proliferation in the wing pouch as well as in the hinge region (figures 1 and 2*e,k*; electronic supplementary material, figure S5E). To explain this, we used the G-TRACE system and found *sd*-mediated GFP expression in the entire wing disc (electronic supplementary material, figure S6), suggesting *sd* is expressed in the entire wing disc at an early larval stage [32].

Together, these results indicate that Sna regulates Hippo signalling-mediated Yki-dependent tissue growth and cell proliferation, most likely downstream of Yki.

### 2.2. Loss of *sna* suppresses Hippo signalling-mediated target gene expression

**Figure 3.**
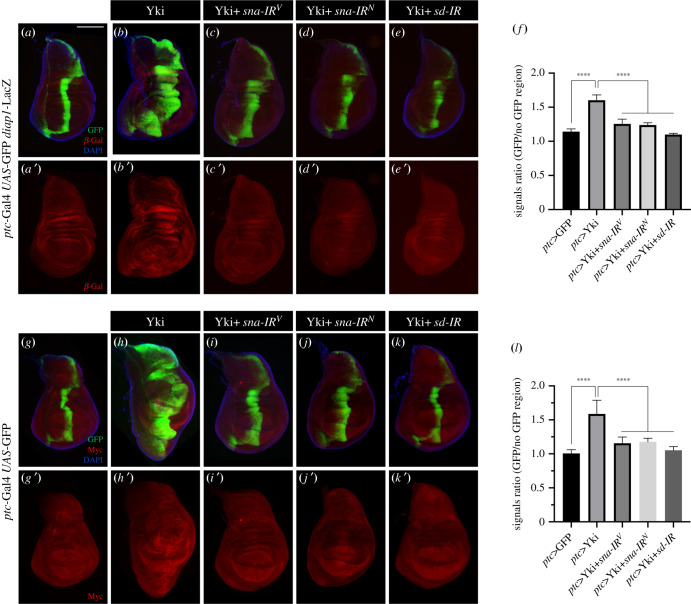
Sna is required for Yki-triggered target gene activation. Fluorescence micrographs of third-instar larval wing discs stained with anti-*β*-Gal antibody (*a*–*e*) or anti-dMyc antibody (*g*–*k*) are shown. Compared with the *ptc* > GFP controls (*a*,*g*), expression of Yki activates Hippo pathway reporter *diap1*-LacZ (*b*) and Myc (*h*), both are suppressed by the depletion of *sna* (*c*,*d*,*i*,*j*) or *sd* (*e*,*k*). (*f*,*l*) Quantification of average signal ratios for GFP region/non-GFP region. (*f*) Left to right: *n* = 3, *n* = 3, *n* = 4, *n* = 3, *n* = 5. (*l*) Left to right: *n* = 5, *n* = 5, *n* = 6, *n* = 7, *n* = 5. One-way ANOVA was used to compute *p*-values, ****p < 0.0001. Scale bar: 100 µm in (*a*–*e*), (*g*–*k*).

To verify the role of Sna in regulating Hippo signalling, we checked the expression of Hippo pathway target genes, including *diap1* and *myc*, which are required for cell survival and proliferation, respectively [33,34]. Compared with the controls, the overexpression of Yki driven by *ptc*-Gal4 resulted in upregulated expression of *diap1*-LacZ and Myc [35], which were suppressed by depleting *sna,* while *sd-IR* served as a positive control (figure 3). Moreover, upregulated *diap1* expression in Yki overexpression clones was suppressed by depleting *sna* (electronic supplementary material, figure S7), confirming that Sna is required for Yki-triggered target gene expression.

### 2.3. Sna is necessary and sufficient for tissue growth

**Figure 4.**
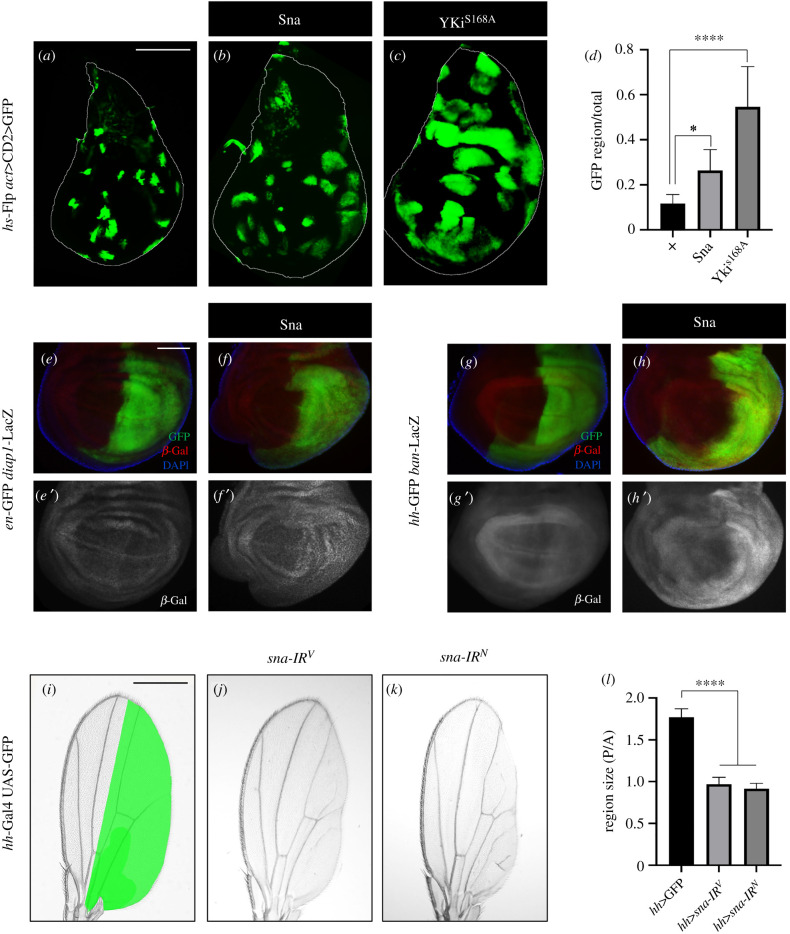
Sna promotes Yki target gene expression and tissue growth. Fluorescence micrographs of third-instar larval wing discs with clones (marked by GFP) (*a*–*c*), stained with anti-*β*-Gal antibody (*e*–*h*) are shown. GFP-labelled Sna (*b*) or Yki^S168A^ (*c*) overexpression clones are larger than wild-type controls (*a*). (*d*) Quantification of clone size/total size shown in (*a*–*c*) (*n* = 8, *n* = 10, *n* = 11). One-way ANOVA was used to compute *p*-values, ****p < 0.0001, *p < 0.05. Expression of Sna activates Hippo reporter *diap1*-LacZ (*f*) and *ban*-LacZ (*h*), compared with the controls (*e*,*g*). (*i*–*k*) Light micrographs of *Drosophila* adult wings are shown. Compared with the *hh*-Gal4 control (*i*), the depletion of *sna* reduces the size of posterior compartment (*j*,*k*). (*l*) Statistical analysis of the adult wing size (P/A) is shown (*n* = 14, *n* = 11, *n* = 12). One-way ANOVA was used to compute *p*-values, ****p < 0.0001. Scale bar: 100 µm in (*a*–*c*), 50 µm in (*e*–*h*), 250 µm in (*i*–*k*).

To test whether Sna is sufficient to promote tissue growth, we generated Flp-out clones that express UAS-transgenes by *act*-Gal4. Compared to the control, the expression of Sna resulted in a mild increase of clonal size, while the expression of the activated Yki^S168A^, which was included as a positive control, caused dramatic overgrowth of the clones (figure 4*a*–*d*). In addition, Sna expression along the A/*P* boundary driven by *ptc*-Gal4 induced a mild overgrowth in the hinge region (electronic supplementary material, figure S8A,B), accompanied by upregulated expression of *diap1* (electronic supplementary material, figure S8A’,B’). Furthermore, ectopic Sna expression in the P compartment of wing discs activated the transcription of *diap1* (figure 4*e,f*) and *ban* (figure 4*g,h*), another Hippo pathway target gene [36]. Together, these data suggest that ectopic Sna is sufficient to activate the expression of Hippo pathway target genes and promote tissue growth.

To further investigate the physiological function of Sna in development, we first checked the endogenous expression of *sna* in the wing discs. To this end, we used a Sna-GFP reporter, which carries a genomic fragment in which Sna has been fused in-frame at its C-terminus to GFP. We found that Sna is ubiquitously expressed in the third-instar wing discs (electronic supplementary material, figure S9A), and its expression in the *p*-compartment was significantly reduced upon *hh-*Gal4 driven *sna-IR* expression (electronic supplementary material, figure S9B,C). Next, we checked whether *sna* is required for normal wing growth. As *ptc > sna-IR* did not notably affect cell proliferation and tissue growth along the A/P compartment boundary in the developing wings (electronic supplementary material, figure S3), presumably due to the relative mild expression of the *ptc*-Gal4 driver, we raised *ptc > sna-IR* animals at 29°C to increase the Gal4 activity and observed reduced sizes of the L3–L4 area (electronic supplementary material, figure S10). Moreover, *sna* depletion in the P compartment of wing discs by *hh*-Gal4, a stronger Gal4 driver, resulted in diminished posterior areas in the adult wings (figure 4*i*–*l*), suggesting that *sna* is physiologically required for proper tissue growth in normal development.

### 2.4. Sna physically interacts with Scalloped to promote Scalloped-dependent cell proliferation

**Figure 5.**
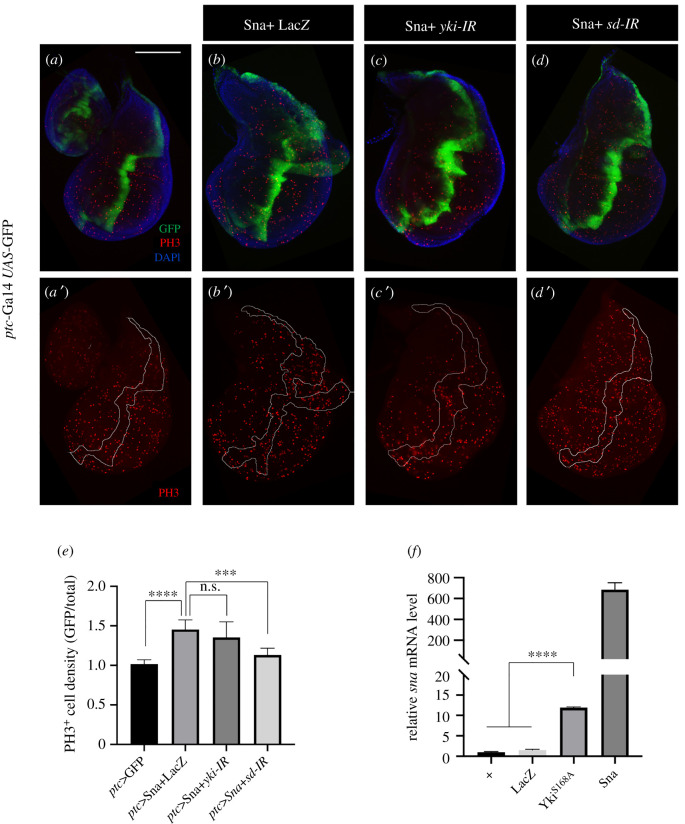
Sna promotes Sd-dependent cell proliferation. (*a*–*d*) Fluorescence micrographs of third-instar larval wing discs stained with anti-pH3 antibody are shown. Compared with the control (*a*), the expression of Sna driven by *ptc*-Gal4 promotes cell proliferation (*b*), which is suppressed by the depletion of *sd* (*d*), but not that of *yki* (*c*). (*e*) Quantification of pH3-positive cell density ratio for GFP region/total region is shown (*n* = 7, *n* = 11, *n* = 8, *n* = 6). (*f*) Histogram showing the levels of *sna* mRNAs as measured by RT-qPCR. Error bars represent standard deviation from three independent experiments. One-way ANOVA was used to compute *p*-values, ****p < 0.0001, ***p < 0.001, n.s. indicates not significant. Scale bar: 100 µm in (*a*–*d*).

Since Sna is required for Yki-triggered tissue overgrowth, cell proliferation and target gene expression (figures 2*g*–*j* and 3; electronic supplementary material, figure S5), we reasoned that Sna might act downstream of Yki. In agreement with this hypothesis, Sna-induced cell proliferation remained unchanged upon *yki* depletion (figure 5*a*–*c*,*e*), while *sna* transcription was upregulated by activated Yki (figure 5*f*). Together, these results indicate that Yki activates the expression of Sna, which acts as a downstream mediator of Yki activity.

**Figure 6.**
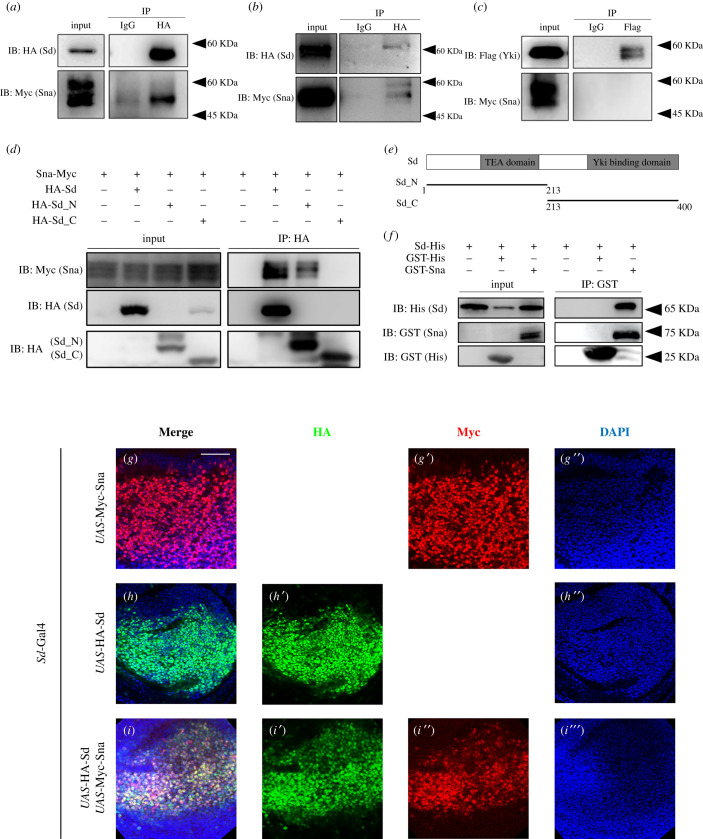
Sna physically interacts with Sd. Co-IP experiment showing that Sna physically interacts with Sd (*a*), but not Yki (*c*) in S2R+ cells. Co-IP experiment showing that Sna interacts with Sd *in vivo* (*b*)*.* Co-IP experiment analysis showing that Sna interacts with Sd_N, but not Sd_C (*d*). Diagram of Sd protein and truncated fragments (*e*). GST pull-down assay showing that Sna directly binds to Sd (*f*). (*g*–*i*) Fluorescence micrographs of subcellular localization of HA-Sd and Myc-Sna are shown. HA-Sd and Myc-Sna expression in the wing pouch are driven by *Sd*-Gal4. Scale bar: 50 µm in (*g*–*i*).

The Hippo-Yki signalling modulates tissue growth through the transcription factor Sd [5,37], which has been reported to regulate transcription via interacting with a wide range of cofactors, including Yki [38]. For instance, the transcription repressor Nerfin-1 antagonizes the activity of Yki-Sd complex by directly binding to Sd [39]. To investigate a possible interaction between Sna and Sd, we first examined whether Sna-induced cell proliferation depends on Sd. We found *ptc* > Sna-triggered cell proliferation was significantly suppressed by knockdown of *sd* (figure 5*d*–*e*), in contrast with that of *yki* (figure 5*c*), suggesting Sna promotes Sd-dependent cell proliferation. Next, we performed Co-IP assay and confirmed that Sna physically interacts with Sd both in *Drosophila* S2R+ cells (figure 6*a*) and *in vivo* (figure 6*b*). Intriguingly, the molecular weight of HA-Sd *in vivo* appears higher than that in S2R+ cells, implying a possible post-translational modification of Sd *in vivo* [40,41]. By contrast, Sna failed to interact with Yki (figure 6*c*). In addition, Sna interacts with the N-terminal half of Sd, but not its C-terminal part (figure 6*d,e*). Furthermore, GST pull-down assay indicated that Sna directly binds to Sd (figure 6*f*). Finally, Sna co-localizes with Sd in the nucleus of the wing pouch (figure 6*g*–*i*). Together, these results indicate that Sna might act as a transcriptional cofactor of Sd to promote Sd-dependent cell proliferation.

## 3. Discussion

Snail (Sna) belongs to the Snail superfamily of C_2_H_2_-type zinc finger proteins [42], which functions as a transcription factor by binding to the consensus sequence CAGGTG [13]. Sna was first identified in *Drosophila* as a transcription regulator involved in embryonic patterning [14] and was later characterized as a key regulator of EMT and tumour metastasis by repressing E-cadherin expression [43]. However, the role of Sna in normal tissue growth has remained unknown [22]. In this study, we employed *Drosophila* as a model organism to investigate the physiological functions of Sna in tissue growth and revealed that Sna is not only required for impaired Hippo signalling-induced accelerated cell proliferation and tissue overgrowth, but also contributes to proper tissue growth in normal development. Our genetic epistasis analysis showed that the loss of *sna* suppressed *hpo* or *wts* depletion, or Yki overexpression-induced cell proliferation and tissue overgrowth, whereas ectopic Sna-induced cell proliferation was not suppressed by *yki* depletion, suggesting Sna acts downstream of Yki to regulate Hippo signalling-mediated tissue growth and cell proliferation. Consistently, *sna* expression is upregulated by Yki, which provides a molecular explanation for the above genetic data. Moreover, Sna forms a transcriptional complex with Sd by direct physical interaction and promotes Sd-dependent cell proliferation.

In support of our findings, ectopic Sna has previously been shown to activate the expression of dIAP1 and Myc, both of which are targets of Yki, yet the role of Sna in Hippo-Yki signalling was not further investigated in the research [44]. Thus, our study represents the first report that Sna is involved in Hippo signalling-mediated tissue overgrowth and that Sna is also required for normal tissue growth in development. Although we provide evidence here that Sna promotes cell proliferation and tissue growth, previous studies have shown that Sna overexpression also triggers cell death and affects cell size [44,45]. Therefore, as a result of the comprehensive effect of these cellular processes, Sna overexpression promotes a mild growth phenotype, much less prominent than that induced by Yki overexpression (figure 4*a*–*d*). Besides regulating tissue/organ growth and tumour formation, the Hippo pathway is also involved in other functions, including stem cell self-renewal and differentiation [46]. Intriguingly, murine Snail/Slug were reported to form complexes with YAP/TAZ in regulating skeletal stem cell development and functions [21,44], suggesting Sna family members may regulate the Hippo-Yki signalling by distinct mechanisms in a context-dependent manner. Both Sna and Hippo signalling play pivotal roles in tumour progression [47,48]; therefore, this study also shed light on the interaction and underlying mechanism between Sna and Hippo signalling in cancer development.

## 4. Methods

### 4.1. Fly strains

All flies were raised on a standard cornmeal and agar medium at 25°C unless otherwise indicated. Fly strains used in this article have been described previously: *ptc*-Gal4 [49], *en*-Gal4, *hh*-Gal4, *Sd-*Gal4, *UAS*-LacZ, *UAS*-GFP, *UAS-hpo-IR* and *UAS-wts-IR* [50], *UAS*-Yki, *diap1*-LacZ, *UAS*-Sna, *UAS-*Flp *UAS-*RFP *act > y + >* EGFP, *tub*-Gal80^ts^. *ex*-LacZ, *ban*-LacZ, *UAS-sd-IR* and *UAS-*HA-Sd were gifts from professor Lei Zhang. *UAS-sna-IR* (28679) and *UAS*-Yki^S168A^ (28816) were obtained from the Bloomington stock centre, *UAS-sna-IR* (6232), *UAS-yki-IR* (40497) and Sna-GFP (318402) were obtained from the Vienna *Drosophila* RNAi Center, *UAS-sna-IR* (3956R-5) was obtained from Japanese National Institute of Genetics (NIG). Transgenic flies expressing *UAS*-Myc-Snail was generated by standard P element-mediated transformation.

To induce Flp-out clones, animals were reared at 25°C for 3 days, heat-shocked at 37°C for 15 min and recovered at 29°C for 2 days prior to dissection. To obtain the *hh* > *sna-IR* wing phenotype, animals were raised at 29°C to enhance the Gal4 activity. For *ptc* > Sna experiments, animals were raised at 20°C to avoid ectopic Sna-induced larval lethality. When *tub*-Gal80^ts^ was used to regulate Sna expression, animals were raised at 25°C for 3 days, then shifted to 29°C for 2 days before dissection.

### 4.2. Immunostaining

Antibody staining was performed by standard procedures for third-instar larval imaginal discs. Primary antibodies included rabbit anti-Phospho-Histone H3 (1 : 400, Cell Signaling Technology, CST, cat. no. 9701), mouse anti-*β* Gal (1 : 500, Developmental Studies Hybridoma Bank, DSHB, cat. no. 40–1a), rabbit anti-Myc (1:500, Santa Cruz Biotechnology, d1-717), rabbit anti-Cleaved Caspased-3 (1 : 400, CST, cat. no. 9661), mouse anti-Myc-Tag (1 : 100, CST, cat. no. 2276), rabbit anti-HA-Tag (1 : 100, CST, cat. no. 3724) and mouse anti-GFP (1 : 200, Roche, cat. no. 11814460001). Secondary antibodies were goat anti-rabbit CY3 (1 : 1000, Life technologies, cat. no. A10520), goat anti-mouse CY3 (1 : 1000, Life Technologies, cat. no. A10521) and goat anti-rabbit Alexa Flour 488 (1 : 1000, Life Technologies, cat. no. A32731).

### 4.3. Image and quantification of fly wings

Wings were dissected and placed on slide with alcohol/glycerol (1 : 1) medium. Light images of wing were taken by Olympus BX51 microscope. Adobe Photoshop 2020 was used to retouch the images.

### 4.4. Reverse transcription polymerase chain reaction

For heat shock experiment, animals were raised at 25°C, heat-shocked at 37°C for 30 min and recovered at 29°C for 2 h before experiments. Total RNAs were isolated from whole third-instar larvae.

For *hh* > *sna-IR* experiments, animals were raised at 25°C. Total RNAs were isolated from the wing disc of third-instar larvae, and RT-qPCR was performed as previously described. *rp49* served as the internal control.

Primers used are provided:

*rp49-*FP: TACAGGCCCAAGATCGTGAA

*rp49-*RP: TCTCCTTGCGCTTCTTGGA

*sna-*FP: ATGGCCGCCAACTACAAAAG

*sna-*RP: GCAAACTGTGAGTCCTTGGTC

### 4.5. Co-Immunoprecipitation

*Drosophila* S2R+ cells were cultured in Corning Insectagro DS2 with 10% FBS (HyClone). Effectene Transfection Reagent (Qiagen) was used for co-transfection of Plasmids pUAST-Flag-Yki, pUAST-Myc-Sna, pUAST-HA-Sd, pUAST-HA-Sd_N, pUAST-HA-Sd_C and Actin-GAL4 as indicated. Cells were lysed in RIPA buffer with PMSF 48 h after transfection and proceeded with the standard co-immunoprecipitation protocols.

For *in vivo* co-immunoprecipitation experiment, third-instar larval wing discs over-expressing *UAS*-Myc-Sna and *UAS-*HA-Sd driven by *Sd-*GAL4 were used. Tissues were lysed in RIPA buffer with PMSF and proceeded with the standard co-immunoprecipitation protocols.

Antibodies used in this study were as follows: rabbit anti-HA (CST, cat. no. 3724), mouse anti-HA (CMCTAG, cat. no. AT0024), normal rabbit lgG (CST, cat. no. 2729), mouse anti-Myc (CST, cat. no. 2276), rabbit anti-flag (CST, cat. no. 14793), mouse anti-flag (Sigma, cat. no. F3165), goat anti-rabbit IgG (Abways, cat. no. AB0101) and goat anti-mouse IgG (Abways, cat. no. AB0102).

### 4.6. GST pull-down assays

Plasmids for Sd-His, GST-Sna and GST-His were transfected into *E. coli*. Bacterial cell lysates were prepared as described [51]. Pierce GST Protein Interaction Pull-Down Kit (Thermo 21516) was used for pull down and analysed by western blot. Antibodies used in this study were as follows: rabbit anti-His (CST, cat. no. 2365), rabbit anti-GST (Rackland, cat. no. 24833) and goat anti-rabbit IgG (Abways, cat. no. AB0101).

## Data Availability

The main data supporting this work are available within the article and additional data are provided in the electronic supplementary material.
